# Selection of *Fusarium* Trichothecene Toxin Genes for Molecular Detection Depends on TRI Gene Cluster Organization and Gene Function

**DOI:** 10.3390/toxins11010036

**Published:** 2019-01-14

**Authors:** Ria T. Villafana, Amanda C. Ramdass, Sephra N. Rampersad

**Affiliations:** Department of Life Sciences, Faculty of Science and Technology, The University of the West Indies, St. Augustine, Trinidad and Tobago; riatvill@hotmail.com (R.T.V.); ac_ramdass@hotmail.com (A.C.R.)

**Keywords:** *Fusarium*, molecular detection, mycotoxin, trichothecenes

## Abstract

Food security is a global concern. *Fusarium* are among the most economically important fungal pathogens because they are ubiquitous, disease management remains a challenge, they produce mycotoxins that affect food and feed safety, and trichothecene mycotoxin production can increase the pathogenicity of some *Fusarium* species depending on the host species. Although trichothecenes may differ in structure by their patterns of hydroxylation or acetylation, these small changes have a significant impact on toxicity and the biological activity of these compounds. Therefore, detecting and identifying which chemotype is present in a given population are important to predicting the specific toxins that may be produced and, therefore, to evaluating the risk of exposure. Due to the challenges of inducing trichothecene production by *Fusarium* isolates in vitro for subsequent chemical analysis, PCR assays using gene-specific primers, either singly or in combination, designed against specific genes of the trichothecene gene cluster of multiple species of *Fusarium* have been developed. The establishment of TRI genotypes that potentially correspond to a specific chemotype requires examination of an information and knowledge pipeline whose critical aspects in sequential order are: (i) understanding the TRI gene cluster organization which differs according to *Fusarium* species under study; (ii) knowledge of the re-arrangements to the core TRI gene cluster over evolutionary time, which also differs according to *Fusarium* species; (iii) the functions of the TRI genes in the biosynthesis of trichothecene analogs; and (iv) based on (i)–(iii), selection of appropriate target TRI gene(s) for primer design in PCR amplification for the *Fusarium* species under study. This review, therefore, explains this pipeline and its connection to utilizing TRI genotypes as a possible proxy to chemotype designation.

## 1. Introduction

Understanding the complex interactions of how and why *Fusarium* mycotoxins are produced will provide critical information for developing novel strategies to reduce the incidence and severity of *Fusarium* diseases and mycotoxin contamination of human food and animal feed. Part of the difficulty in characterizing these mycotoxins lies in the species-specific toxico-kinetics, bioavailability and the mechanisms of action discovered in both in vitro and in vivo studies [1,2,3]. Concerted efforts to mitigate this global threat to food safety caused by mycotoxin-producing *Fusarium* species are reflected in the cumulative work of over 100 active research laboratories worldwide, some for over 25 years [4,5,6].

**Figure 1:**
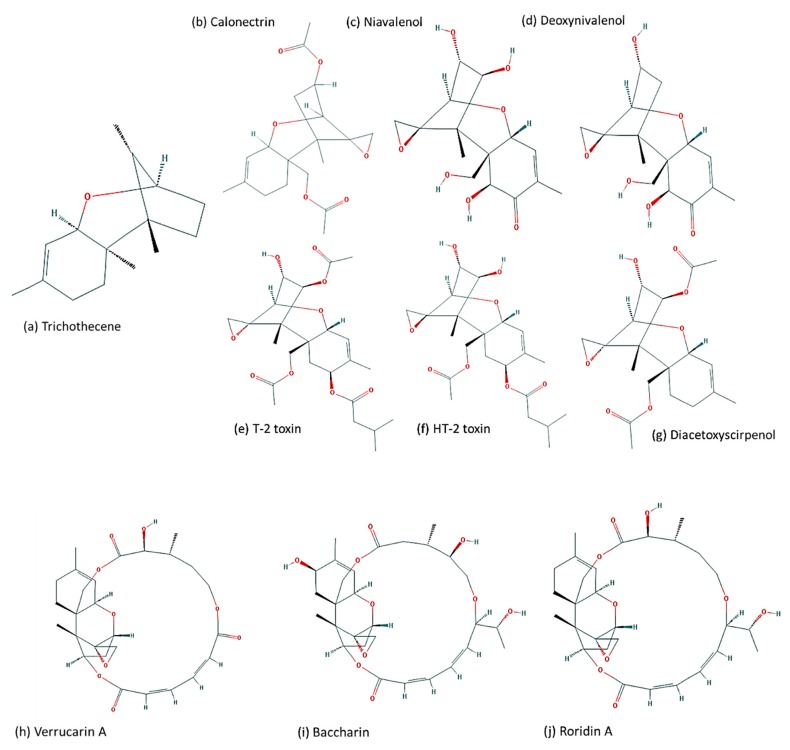
Structural diversity and complexity of trichothecene analogs; (**a**–**g**) illustrate the simpler trichothecene structures; (**h**–**j**) illustrate the more complex macrocyclic structures which are considered to be the more toxic trichothecenes. All figures were retrieved from the open chemistry database, PubChem [https://pubchem.ncbi.nlm.nih.gov/compound/21117948].

Trichothecenes are among the most economically important mycotoxins and have a significant impact on cereal and grain production [7]. Mycotoxin contaminated grains pose a health risk for human consumption, animal feed or malting purposes [5]. In the US, based on a report from the FDA, a conservative estimate of annual loss due to mycotoxin contamination ranges from USD 0.5 million to over USD 1.5 billion from aflatoxin (corn and peanuts), fumonisin (corn), and deoxynivalenol (wheat) [8]. Trichothecenes inhibit protein translation and cause oxidative stress in eukaryotes [9]. In plants, trichothecenes produced by *Fusarium* pathogens (e.g., DON, DAS and T-2-toxin) cause phytotoxicity and aid in increased pathogenicity in several crop hosts [10,11,12]. More than 200 different trichothecenes and trichothecene derivatives have been isolated [13,14]. Trichothecene structures are highly diverse and complex for two main re6asons: (i) variation in the assembly of the 15-carbon skeleton that comprises the backbone of all sesquiterpenes; and (ii) variation in the functional groups that bind to the core structural scaffold in specific regio- and stereospecific manners [15]. As a result, trichothecenes can be classified into four groups based on the pattern of substitution of tricyclic 12,13-epoxytrichothec-9-ene (EPT) (see the review by McCormick et al. [16]), which results in a scheme of four types: A, B, C and D (Figure 1a–g). Type D trichothecenes have more complex structure due to an additional ring linking the C-4 and C-15 position (e.g., roridin A, verrucarin A and baccharin). This feature makes them macrocyclic (Figure 1h–j). Macrocyclic trichothecenes are considered more toxic than the simpler structured trichothecenes [16]. The most recent BIOMIN World Mycotoxin Survey (https://www.biomin.net/en/biomin-mycotoxin-survey/) [17], which investigates the incidence of six major mycotoxins in the agricultural commodities used for livestock feed from 81 countries worldwide, reported that, singly, deoxynivalenol (DON) was the most abundant mycotoxin worldwide and was found in 81% of all samples, followed by fumonisins, which were found in 71% of samples. Type A trichothecenes, e.g., DAS and T-2 toxin, are considered more cytotoxic than Type B trichothecenes, e.g., DON. Chemotypes of Type B trichothecene-producers have been described for *Fusarium* species as: chemotype I are DON-producers and/or its acetylated derivatives; and chemotype II are NIV and/or 4-ANIV-producers [18]. The DON chemotype can be further split into chemotype IA, which are 3-ADON-producers, and chemotype IB, which are 15-ADON producers [19].

One of the major analytical challenges to chemical detection, identification and quantification of trichothecenes is their structural complexity and differing levels of toxin production. PCR-based genotyping of the TRI gene cluster as a molecular approach to chemotype prediction has been proposed for a range of *Fusarium* species. To meet a research objective to detect TRI gene(s) for the establishment of genotypes that potentially correspond to a specific chemotype, an information and knowledge pipeline must be developed and the critical aspects of this pipeline to be examined in sequential order are: (i) understanding the TRI gene cluster organization which differs according to *Fusarium* species under study; (ii) knowledge of the re-arrangements to the core TRI gene cluster over evolutionary time, which also differs according to *Fusarium* species; (iii) the functions of the TRI genes in the biosynthesis of trichothecene analogs; and (iv) based on (i)–(iii), selection of appropriate target TRI gene(s) for primer design in PCR amplification for the *Fusarium* species under study. This review, therefore, explains this pipeline and its connection to utilizing TRI genotypes as a proxy to chemotype designation.

## 2. Biosynthetic Gene Clusters

To design molecular tools for detecting genes that are involved in the biosynthesis of secondary metabolites, it is important to understand the arrangement and evolutionary maintenance of those genes that encode proteins of the same metabolic pathway. Genes involved in the synthesis of secondary metabolites are usually arranged in clusters and their expression is co-regulated in fungi [20,21,22]. Mycotoxin genes are expressed as a biosynthetic gene cluster (BGC). A BGC can be defined as a group of two or more genes physically located as a cluster in a specific location in the genome [23]. These BGCs usually consist of genes that encode polyketide synthases (PKS), non-ribosomal peptide synthetases (NRPS), hybrids (PKS-NRPS), terpene cyclases (TCs) and prenyltransferases (PTs) in addition to adjacent genes that assist in regulation of gene expression and in transport of the metabolites [24,25]. There are also genes that encode enzymes that serve to modify the parent structure including acyltransferases, amino transferases, dehydrogenases, reductases, dioxygenases, methyltransferases, monooxygenases, and prenyltransferases [26].

Toxic secondary metabolites, such as trichothecenes, are families of structural or chemical analogs that share a common core structure, but differ in the presence of different functional groups that may be attached to this core structure [21,27]. These structural variations may be determined by the gain, loss or differences in function of specific genes that encode modifying enzymes [26]. BGC families encoding the production of polyketides, as in the case of mycotoxins, evolve in a family-specific manner, and are impacted by internal recombination and physical re-positioning of specific genes within the cluster [23,26]. Comparisons of gene cluster organization governing trichothecene production indicate that this is also the case for *Fusarium*. The development of different gene cluster arrangements in different species of *Fusarium* suggest that BGC modifications may confer an evolutionary advantage. This may explain why gene clusters, for the expression of mycotoxins as secondary metabolites, are maintained in the genome [21]. For example, the trichothecene NIV, but not DON, is required for increased aggressiveness of *Fusarium* on maize but not on wheat [28]. If increased pathogenicity reflects an increased fitness advantage, then chemotype diversity may be maintained as an adaptation to different hosts. Further, synteny of genes encoding proteins of mycotoxin production in fungi may represent: (i) a signature of positive selection for protection against the accumulation of toxic intermediates during biosynthesis; and (ii) the route of evolution of the biosynthetic pathways may have developed as stepwise modifications of toxic intermediates [29].

## 3. Trichothecene Gene Cluster Structure and Organization

**Figure 2:**
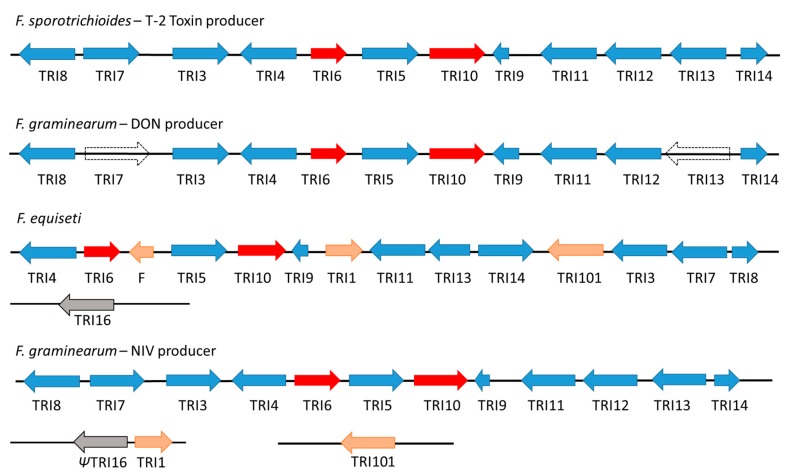
Comparison of the proposed trichothecene biosynthetic gene cluster of *Fusarium sporotrichioides*, *F. graminearum*, and *F. equiseti* [1,30,34,36,37,43].

Formulating a strategy to characterize trichothecene genotypes and the possible relationship to an isolate’s chemotype requires knowledge of how trichothecene (TRI) genes are arranged as a BGC. The trichothecene biosynthetic gene (TRI) cluster is one of the most extensively studied secondary metabolite gene clusters in fungi [26]. In *F. graminearum* and *F. sporotrichioides*, the trichothecene gene cluster has been identified as a 26-kb length of DNA that consists of three loci: a single-gene TRI101 locus, a two-gene TRI1-TRI16 locus, and a 12-gene core TRI cluster (Figure 2) [30]. Conventional names of each trichothecene biosynthetic gene comprise three uppercase letters (TRI, trichothecene) and a number relating to the gene (e.g., TRI3 and TRI4), according to *Fusarium* genetic nomenclature [31,26]. These genes function in the biosynthesis, regulation, and transport of mycotoxins across the plasma membrane [16,32]. Brown et al. [33] sequenced 14–16 kb of DNA that flanked the TRI gene core cluster and, although 12 new ORFs in both *F. sporotrichioides* and *F. graminearum* species were identified, gene expression and gene deletion studies indicated that these flanking regions were not required for trichothecene biosynthesis, thereby defining the 5′ and 3′ ends of the core TRI gene cluster. Seven genes (TRI8, TRI7, TRI3, TRI4, TRI5, TRI11, and TRI13) encode enzymes that catalyze 10 reactions in the biosynthesis of trichothecene [34]. Protein annotations for TRI genes identified for *F. graminearum* PH-1 isolate (assembly ASM24013v3) on GenBank and the most recent annotations by Proctor et al. [30] are given in Table 1. The smaller, separate two-gene locus consisting of TRI1 and TRI16 is supposedly non-functional in *Fusarium* species but reports indicate that they have been relocated to the core TRI gene cluster of *F. equiseti* [30]. TR101 was also inserted into the core cluster of *F. equiseti*, which is indicative of gene rearrangement during the course of evolutionary time. Figure 2 illustrates the proposed trichothecene biosynthetic gene cluster of *F. sporotrichioides, F. graminearum*, and *F. equiseti* [30,34,35,36,37]. Gene TRI6, which recognizes the “TNAGGCC” motif, and TRI10 encode two transcription factors that serve to regulate transcription of TRI genes [26,38,39]. Both genes, therefore, regulate production of trichothecene mycotoxins [26,37,40]. There were no gene rearrangements as shown by the conserved sequential order of genes and the same direction of each arrowhead (which depicts direction of transcription) for each gene for *F. sporotrichioides* and *F. graminearum*. There can be loss of TRI13 gene or loss of TRI7 and TRI13 genes for *F. graminearum* depending on the isolate [26]. Both genes are considered to be pseudogenes (*Ψ*TRI7 and *Ψ*TRI13) where they have lost the ability to produce a functional protein due to mutation accumulation over evolutionary time [26]. Loss of these genes may affect what type of trichothecene mycotoxin is produced, e.g., loss or disruption of TRI7 and TRI13 results in 3-ADON or 15-ADON mycotoxin production [35,37,41]. The TRI13 gene encodes trichothecene-15-O-acetyltransferase (15-ADON), which is involved in production of NIV-producing chemotypes, and disruption of TRI13 gene results in DON-producing chemotypes [36]. Alexander et al. [42] also reported that differential activity of the esterase enzyme encoded by TRI8 determines the 3-ADON and 15-ADON chemotypes in *F. graminearum*.

There are no gene rearrangements, as shown by the conserved sequential order of genes and the same direction of each arrowhead for each gene for *F. sporotrichioides* and *F. graminearum*, which depicts direction of transcription of each gene. There can be loss of one gene (TRI13) or two genes (TRI7 and TRI13) for *F. graminearum* (white arrows). Loss of these genes may affect what type of trichothecene mycotoxin is produced, e.g., loss of TRI7 or TRI13 results in 3-ADON or 15-ADON mycotoxin production. In *F. equiseti*, gain of TRI1 and TRI101 gene and change in relative positions of TRI genes (TRI3, TRI7 and TRI8 are also in the opposite orientation and located at the opposite end of the cluster) results in a different gene organization for this species compared to *F. sporotrichioides* and *F. graminearum*. Another difference is the insertion of Gene F, which is located between TRI5 and TRI6 in *F. equiseti* NRRL 13405. *Ψ*TRI16 indicates TRI16 pseudogene. The red arrows for TRI6 and TRI10 indicate that the orientation, presence of these genes and the location of these genes on the 5′ and 3′ end of TRI5 gene are maintained in the core cluster regardless of species. Genes that flank the TRI gene cluster are not shown.

Traditionally, the chemotype of a fungal species is defined as its profile of natural products or its chemical phenotype i.e., the “chemical expression of a genotype” [45]. Different chemical constituents make up the functional groups to which chemotypes belong. Examples of trichothecene chemotypes are the nivalenol (NIV) - and deoxynivalenol (DON)-producing strains of *Fusarium*. Chemotypes are usually identified by chemical analysis after the toxin is produced in vitro. Mapping the chemotypes of a given *Fusarium* population is important for: (i) understanding the epidemiology the population in a given area; (ii) predicting the risk of contamination and subsequent exposure to a certain chemotype; and (iii) developing preventive models to decrease risk. However, incongruence between relative aggressiveness/pathogenicity to a particular host species and chemotype means that chemotype alone should not be the sole characteristic of a given toxicogenic fungal population [46].

A range of factors affect mycotoxin production in vitro either singly or in combination [47], and include nitrogen and carbon nutrient conditions [47], pH and temperature [48,49,50], oxidative stress [51], water potential [52,53], other abiotic stresses [54], and chemotype [51]. As a result, in vitro incubation conditions directly influence mycotoxin production, which can lead to false negative results [44]. For example, *F. graminearum sensu stricto* produces limited quantities of trichothecenes in liquid media and acetyl derivatives of DON are predominantly produced rather than DON [35,55]. Conversely, field-infected grain test positive for DON with smaller amounts of 3-ADON or 15-ADON [56]. Some isolates simply do not produce any toxin regardless of modifications to laboratory conditions [57]. Lowe et al. [5] described detailed protocols for induction of Type B trichothecene mycotoxins in liquid culture (for small- or large-scale production) in order to detect deoxynivalenol (DON) and its various acetylated derivatives but warns of significant differences among replicates. Challenges in mycotoxin induction for chemical analysis have prompted efforts in genotyping based on the TRI gene cluster towards investigating a possible relationship between genotype and chemotype. Molecular approaches involving the use of PCR-based assays for TRI gene detection and identification with TRI gene-specific primers have been developed [46].

Distinct chemotypes appear to be related to gain or loss of specific genes and specific gene rearrangements and the resultant chemotype may not produce a specific toxin or produce a range of toxins with different toxicological properties. It is suggested that loss or gain or alteration of specific genes, which results in differences in core TRI gene structure, may directly or indirectly influence the production of type A or type B trichothecenes in *Fusarium* [28,30,35,41,58]. Gain of TRI1 and TRI101, which exist as a separate cluster to the core TRI cluster, enables *F. graminearum* to produce NIV mycotoxin. Gain of TRI1 and TRI101 genes and a change in relative positions of TRI genes in *F. equiseti* results in a different gene cluster organization compared to *F. sporotrichioides* and *F. graminearum.* Homologs of TRI101 have been identified in trichothecene-producing and non-producing species of *Fusarium* [59,60].

## 4. Evolution of TRI Genes and Production of Trichothecene Analogs

Understanding what factors contribute to positive selection pressure that results in retention of certain TRI genes will improve our ability to better mitigate mycotoxin risk and may also explain the observed patterns of mycotoxin gene cluster evolution. Sequence comparisons of this gene cluster suggest that there is conservation of certain TRI genes, in terms of gene order and orientation, throughout evolutionary time while other genes have been lost or gained within the genus [4]. This information is important to selecting which genes of the TRI gene cluster should be useful targets for attempting to correlate genotype and chemotype across isolates of a given *Fusarium* species or among *Fusarium* species. Comparative genomics suggest that the major drivers of secondary metabolic gene cluster evolution may be accumulation of interspecies polymorphisms, gain or loss of genes, insertion and deletion events, changes in gene cluster expression, and gene rearrangement within gene clusters which may have resulted in activation of different metabolic pathways to produce specific mycotoxins, structural changes in the metabolites as well as changes in metabolite transport [37,61]. Such gain and loss of genes perhaps reflect changes in selection pressure for a particular biological activity conferred by a specific trichothecene analog. Villani et al. [62] compared TRI gene sequence variation for member species of the *Fusarium incarnatum-equiseti* species complex (FIESC), which has been implicated in disease of a number of crop hosts including wheat and bell pepper [63,64]. Proctor et al. [26] suggested horizontal gene transfer (HGT) events and/or lineage sorting over evolutionary time as possible contributors to TRI gene cluster divergence among *Fusarium.*

One of the selection pressures that influence production of trichothecenes by *F. graminearum* is increased pathogenicity of the fungus depending on the host–pathogen interaction. Isolates of *F. graminearum* that produce NIV trichothecene mycotoxin are more aggressive to maize host plants but not to wheat [28]. The difference in aggressiveness may reflect different fitness levels as a result of adaptation to host availability and host species, and which consequently, may represent one of the drivers of structural diversification of trichothecenes [26]. In addition, one particular modification to the core trichothecene structure (C4 oxygenation) appears to have been lost, re-acquired, and then lost again over evolutionary time and which contributed to the genetic divergence of the *Fusarium* TRI gene cluster [26]. This phenomenon may represent variable phenotypic expression as a result of biotic and/or abiotic selection pressure. Proctor et al. [26] also reported that trichothecene structural diversity was not directly attributed to TRI gene duplication and the number of TRI loci among nine fungal genera based on genomic, phylogenetic, and analytical chemistry studies. Other evolutionary processes may be responsible for the structural divergence of trichothecenes produced by these pathogenic fungi. The net effect of the observed TRI gene cluster variation suggests that trichothecene-producers are in a state of “fitness flux” with subsequent alteration of the TRI gene cluster portfolio and chemo-diversity for each population and species, as is the case for a number of other fungal species [61,65]. Comparative studies on TRI gene cluster variability that is conclusively represented by specific chemotypes at the level of the transcriptome and proteome may reveal the evolutionary forces that act upon positive selection mechanisms to enable certain chemotypes to persist in the field, and, consequently, assist in identifying the main factors affecting the quantity and type of mycotoxin contamination in specific crop hosts [66].

## 5. Using *Fusarium* TRI Gene Cluster for the Identification of Trichothecene Genotypes

**Figure 3:**
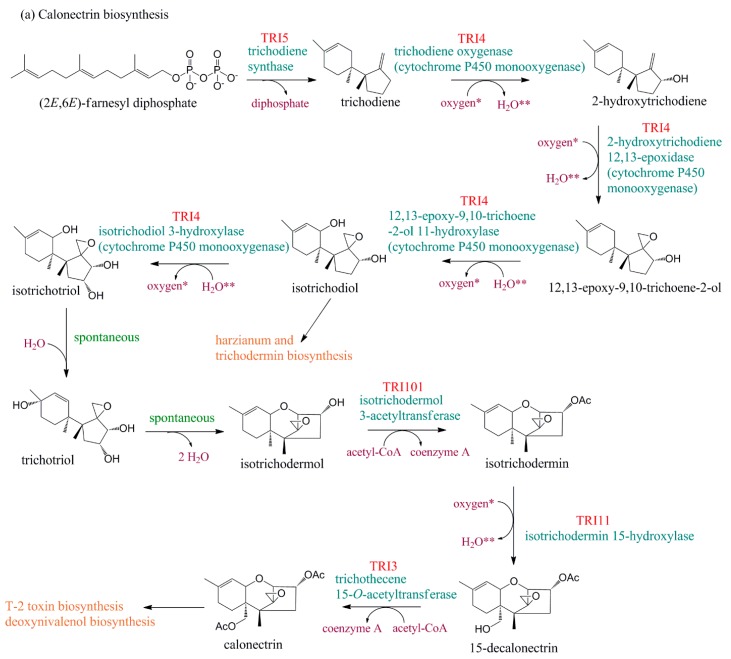

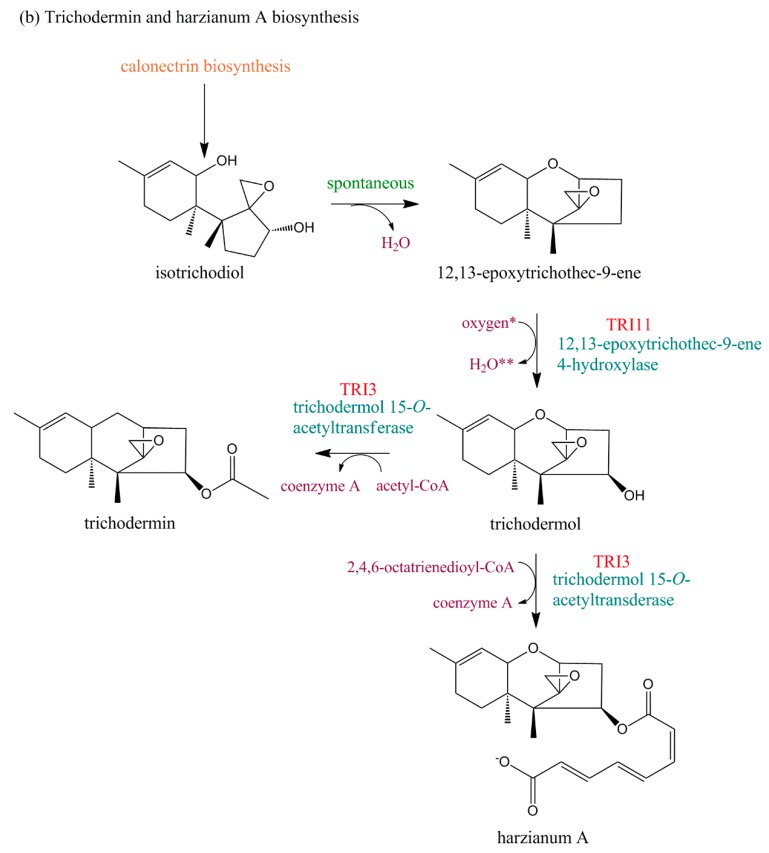

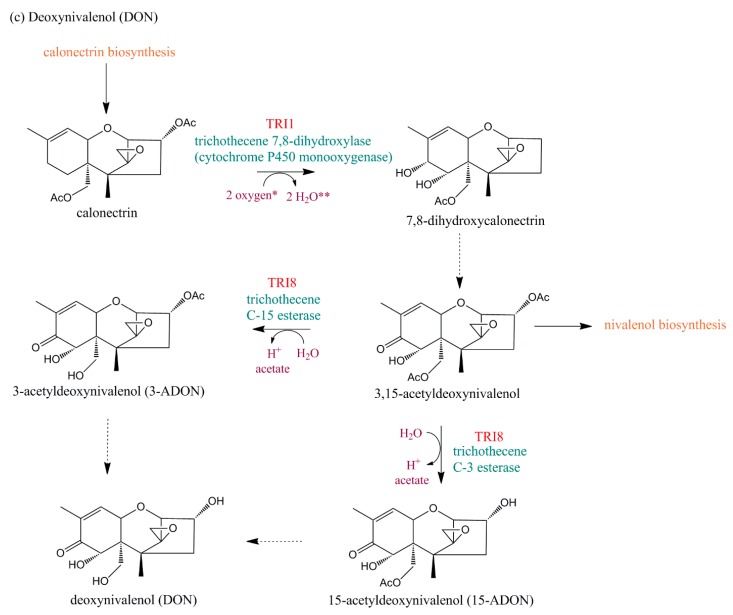

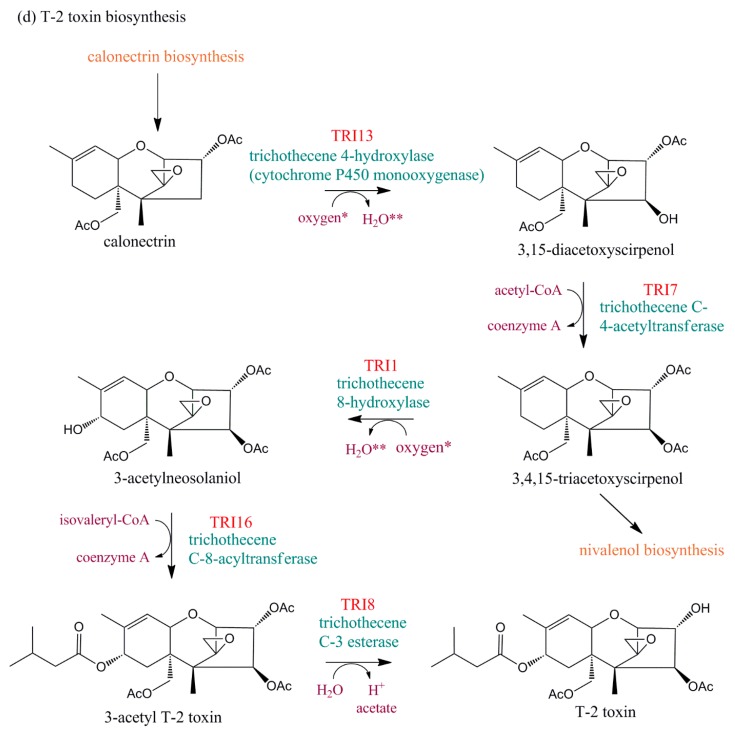

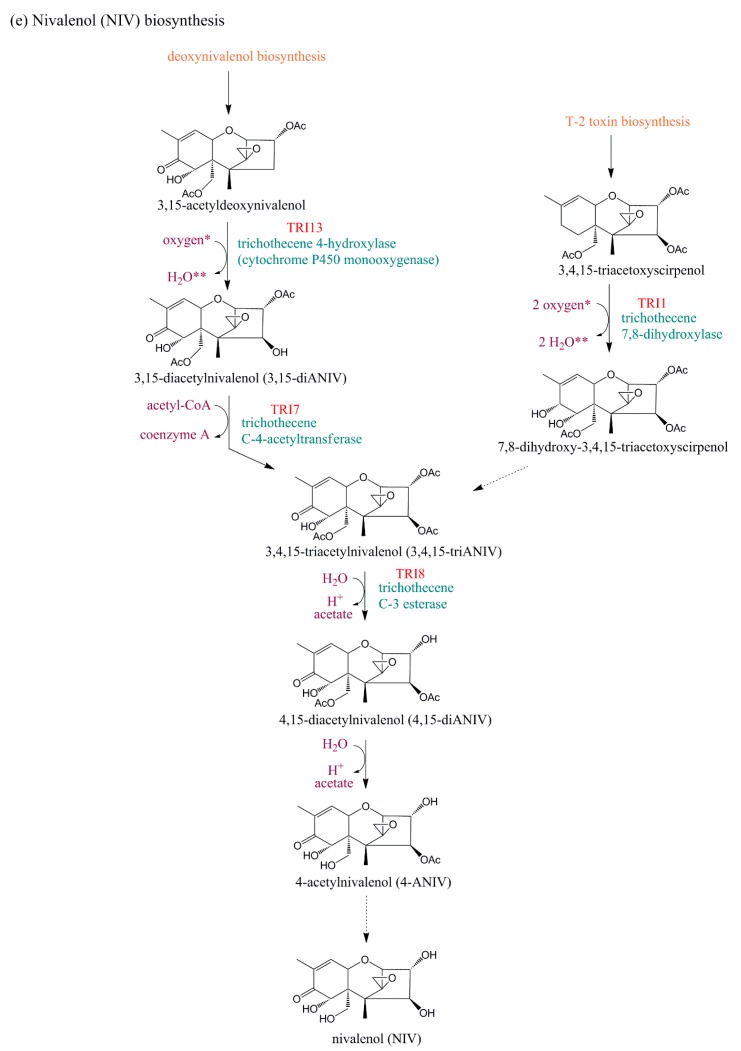
(**a**–**e**) Proposed pathways of trichothecene biosynthesis (pathways were built using ChemBioDraw Ultra software v.12.0). Genes encoding an enzymatic step are identified in red and enzymes that catalyze the reaction are identified in blue. Reactions in which a compound participates as a substrate are indicated in purple. *, a reduced [NADPH-hemoprotein reductase]; **, an oxidized [NADPH-hemoprotein reductase]. Dashed arrows indicate steps for which the gene function remains to be elucidated. Pathways were accessed on the *MetaCyc* Metabolic Pathway public database, super-pathway of trichothecene biosynthesis [https://biocyc.org/META/NEW-IMAGE?type=PATHWAY&object=PWY-7715&detail-level=1] [77].

Understanding the TRI gene cluster composition and arrangement among different *Fusarium* species as well as the subsequent involvement of their encoded proteins in the biosynthesis of different trichothecene analogs have allowed development of a number of PCR-based approaches, using gene-specific primers, as a proxy tool for chemotype prediction [36,46,67,68,69]. However, the use of gene-specific primers can lead to false negative results due to mis-priming or no primer annealing because of indels (insertions/deletions) in the primer annealing sites on template DNA [70,71]. The proposal that presence/absence of a gene in the trichothecene biosynthetic pathway equates to the presence/absence of that secondary metabolite is an over-simplification as synthesis consists of multiple gene products encoded by different genes with multiple gene interactions [71]. Pathways of trichothecene biosynthesis, in terms of the genetics and biochemistry, have been extensively studied in *Fusarium* (Figure 3a–e). Functional analyses of *Fusarium* trichothecene genes have revealed a genetic basis for the observed structural diversity of trichothecene analogs [16,27,42,43]. Research into characterizing the relationship between trichothecene genotype and chemotype in *Fusarium* populations is important for determining population structure, monitoring changes in chemotype as a result of biotic and abiotic influences and, possibly, predicting the ability of isolates to produce trichothecene toxins *in planta* [72,73,74,75]. Such gene interactions may account for the production of isolate-dependent trichothecenes, significant variation in levels of trichothecene production and, in some cases, simultaneous attenuated production of acetylated versions of DON by the same isolates of *F. graminearum* [6,75]. There are also reports of trichothecene production that is not characteristic for a certain TRI genotype [44,76].

### 5.1. Intra- and Interspecies Variation of TRI Genes at the Nucleotide and Amino Acid Sequence Levels

A BLAST comparison of nucleotide and amino acid sequence similarities for each TRI gene in *Fusarium* indicated greater sequence variation *among* species as opposed to variation *within* species, which may indicate intra-specific conservation and usefulness in the design of gene specific primers in PCR-based assays (Table 2). This comparison included only verified *Fusarium* sequences as “PopSets” deposited in NCBI’s GenBank and sequences were retained for comparison if the query coverage > 90% (see Table S1). Among the *Fusarium* species in this study, the lowest nucleotide sequence similarity was found for both TRI4 and TRI14 (75–100%) and the highest nucleotide sequence similarity was found for TRI101 (96–100%). The lowest amino acid sequence similarity was TRI16 (60–100%) and the highest amino acid sequence similarity was TRI11 (90–100%). Comparisons of the nucleotide sequence of the core TRI gene cluster (sequence length = 19277 bases; partial codes for 39 isolates representing 12 different *Fusarium* species; PopSet: 21429250) were also carried out and the results indicated that sequences, with > 90% query coverage, were 91–100% identical for multiple *Fusarium* species. Complete core TRI gene cluster sequences for 17 isolates of *F. graminearum* (GenBank accession numbers MH514940 to MH514957, [78]) were 99–100% identical with 100% query coverage. Sequence identity was reduced to 89% and the query coverage dropped to as low as 15% for species outside of *F. graminearum*. Table 3 details commonly used primers designed against multiple *Fusarium* species in other studies.

### 5.2. Selection of TRI Genes

Based on a comprehensive understanding of the foregoing TRI gene knowledge pipeline, there are a few cases where certain genes of the trichothecene biosynthetic pathway may be suitable for limited gene detection applications by PCR amplification using gene-specific primers. Kimura et al. [68] suggested that TRI genes that may be suitable for studying trichothecene chemotypes (e.g., 4,15-DAS, 3-ADON, and NIV) including those that were inactivated and which resulted in divergence of trichothecene analogs during evolutionary time.

#### 5.2.1. TRI1

Diversification of trichothecene chemotypes in species distant to *F. graminearum* and *F. sporotrichioides* results from functional divergence of the monooxygenase enzymes encoded by TRI1 (Figure 3c–e). Further, the relative location of the TRI1 gene in *F. graminearum* as separate from the main trichothecene gene cluster at a telomere-proximal, which is considered to be a region of high sequence diversity in the genome, compared to the main TRI gene cluster is located at a low diversity genomic position. Rep and Kistler [37] hypothesized that diversification of trichothecenes as T-2 toxin, which differs from NIV and DON chemotype, was potentiated by the change in position of the TRI1 gene near to the telomere. Similar to the cluster itself, TRI1 also exhibits interspecies polymorphisms that appear to reflect chemotype [37]. However, in *F. equiseti*, the TRI1 gene is relocated to the core TRI gene cluster [30]. It is possible that the sequence variation that occurred within the TRI1 gene prior to relocation followed by its subsequent relocation to the core gene cluster resulted in mutation fixation [37].

High nucleotide and amino acid sequence conservation between genes suggest some usefulness in detecting trichothecene producers [45]. However, isolates *within* a particular *Fusarium* species, can share 94% similarity for the same gene. Further, homologs of TRI1 gene can share as little as 85% identity at the amino acid level *between* species (Table 2). As a result, molecular detection of the TRI1 gene was successful for *F. sporotrichioides* but not for *F. graminearum* [45].

#### 5.2.2. TRI5

In a case where genes encode enzymes for reactions occurring at branch points, such genes may be used in trichothecene detection as they are critical to formation of specific end products in the entire biosynthetic pathway [45]. For example, in *Fusarium*, sequence comparisons and gene function analysis revealed that the TRI5 gene encodes terpene cyclase trichodiene synthase which catalyzes the isomerization of farnesyl pyrophosphate to trichodiene which is the first committed step in trichothecene biosynthesis (Figure 3a) [16,34]. Farnesyl pyrophosphate is a precursor for the synthesis of several different classes of metabolites including sterols, terpenes, terpenoids, dolichols, carotenoids, and ubiquinones (UNIPROT, https://www.uniprot.org/uniprot/P14324). In a survey of *Fusarium* species, primers specifically designed to target conserved regions of the TRI5 gene consistently amplified a single amplicon of expected size from trichothecene-producing species but not from non-producing species [81]. Niessen and Vogel [84] also used two conserved regions of the TRI5 gene to design primers that targeted 64 species and varieties of *Fusarium.*

#### 5.2.3. TRI6

TRI6 encodes a transcriptional activator in the core trichothecene biosynthesis gene cluster. TRI6 gene-specific primers designed by Bluhm et al. [85] successfully amplified an expected amplicon for three trichothecene-producing *F. culmorum*, *F. graminearum*, and *F. sporotrichioides*. However, assignment of chemotypes based on TRI6 detection could not be confirmed.

#### 5.2.4. TRI8

Alexander et al. [42] reported that, in *Fusarium*, 3-ADON and 15-ADON are important chemotypes that differ at the intra-specific and inter-specific levels and confirmed the genetic basis for this difference in chemotype based on TRI8-specific primers for genotype characterization of *F. graminearum.* TRI8 from 3-ADON-producers catalyzes deacetylation of the 3,15-diacetyldeoxynivalenol intermediate at carbon 15 to give 3-ADON, whereas TRI8 from 15-ADON-producers catalyzes the deacetylation of 3,15-diacetyldeoxynivalenol at carbon 3 to give 15-ADON (Figure 3c–e). The difference lies in sequence variation of the TRI8 gene, which allows differential expression of TRI8 that results in 3-ADON or 15-ADON chemotypes in *Fusarium*. Comparative analysis of TRI8 gene sequence for 21 *Fusarium* isolates representing multiple species indicated 89–100% nucleotide sequence identity and 76–100% amino acid sequence identity among species (Table 2). However, intra-specific nucleotide sequence variation for *F. graminearum* was low at 99–100% identity.

#### 5.2.5. TRI11

TRI11 encodes the enzyme isotrichodermin C-15 hydroxylase (Figure 3a,b). Wang et al. [82] described a multiplex PCR design involving primers that simultaneously target the TRI11 gene sequences from different chemotype-producing *F. graminearum sensu lato* strains. These primers successfully detected and identified NIV, 3-ADON, and 15-ADON chemotypes using both extracted DNA from fungal isolates and from naturally infected wheat grains. The chemotype-specific DNA pattern reflected the results of chemical analysis for these three trichothecenes. Quarta et al. [80], using TRI11 gene specific primers, confirmed that European isolates of *F. culmorum* that produce deoxynivalenol belong only to the 3-ADON chemotype. Zhang et al. [74] also reported the development of a PCR-based detection method based on TRI11 sequences that could discriminate among NIV, 3-ADON, and 15-ADON producers belonging to *F. asiaticum* species. Talas et al. [86,87] also described the relationship between TRI11 genotype and NIV, 3-ADON, and 15-ADON chemotypes among *F. graminearum* isolates. Comparative analysis of TRI11 gene sequence for 39 *Fusarium* isolates representing multiple species revealed 89–100% nucleotide sequence identity and 90–100% amino acid sequence identity among species (Table 2). Conversely, inter-species variation at the nucleotide sequence for 33 isolates was low at 94–100% identity and even lower for the amino acid sequence at 98–100%. This may explain the usefulness of species-specific primers designed to target conserved regions of this TRI11 gene.

#### 5.2.6. TRI12

Kulik et al. [44] recently reported on the utility of the TRI12 gene for the identification of trichothecene genotypes through next generation sequence (NGS) comparisons in addition to barcode analyses. This approach required generation of whole genome libraries followed by assembly and annotation of TRI12 sequences. Sequence comparisons revealed polymorphisms in the TRI12 gene of *F. graminearum* that appear to contribute to variation at the inter-species level rather than at the intra-species level. A comparison of intra-species nucleotide sequence variation for TRI12 gene was very low at 99–100% identity (Table 2). Sequence polymorphisms that border this gene appear to be related to specific chemotypes which make these regions relevant for the development of degenerate primers in PCR-based detection.

The TRI12 gene was also used for development of qPCR tools for the detection and quantification of NIV, 3-ADON, and 15-ADON producers in single wheat seeds [44]. Nielsen et al. [69] also used qPCR to quantify the same three chemotypes among different seed sets and 378 different grain samples (wheat, barley, triticale, rye and oat) from Denmark. These qPCR data also suggested a historical shift in *Fusarium* species composition in this region.

#### 5.2.7. TRI13

In trichothecene biosynthesis, the gene TRI13 encodes a cytochrome oxidase that catalyzes oxygenation of deoxynivalenol (DON) to produce nivalenol (NIV) (Figure 3d,e). *Fusarium* isolates with a functional TRI13 can produce both DON and NIV, whereas isolates that carry the *Ψ*TRI13 pseudogene produce DON but not NIV. There are deletions that span large areas of the coding regions of the *Ψ*TRI13 gene of *F. graminearum*. These deletions result in a non-functional TRI13 gene. Using allele-nonspecific primers that target these regions generate PCR amplification results in PCR products that differ by 276 bp [71]. Kim et al. [88] reported on a strong and significant correlation between TRI13 genotype and DON and NIV production among 163 trichothecene-producing strains of *F. graminearum* from Korea and the United States.

Molecular discrimination between DON-producing and NIV-producing strains of *F. graminearum* from Nepal was described by Desjardins et al. [71]. For detection of chemically confirmed DON producers, PCR primers that anneal to sites at the ends of the gene are more reliable than primers that bind closer to the indels [83,88]. Reliability of this PCR-based approach is affected by inability of primers to anneal to complementary regions of target sites due to polymorphisms. For example, the *Ψ*TRI13 gene sequence of *F. graminearum* isolates from Nepal is polymorphic at this region [83]. Waalwijk et al. [89] also designed TRI13 gene-specific primers that were able to differentiate between DON and NIV chemotype based on amplicon length difference. However, the TRI13 gene length polymorphism approach was unable to identify 15-ADON-producers of *F. graminearum sensu stricto* [90]. Non-functional pseudogenes accumulate sequence variability at a faster rate when compared to functional genes; thus, suspected TRI13 genotypes require further validation. Comparison of four different *Fusarium* species revealed 92–100% nucleotide sequence similarity and 76–100% amino acid sequence identity among *Fusarium* species (Table 2).

#### 5.2.8. TRI3 and TRI6 in Combination

A multiplex PCR assay was developed for simultaneous identification of the species and 3-ADON, 15-ADON and NIV trichothecene chemotypes for *F. asiaticum* and *F. graminearum sensu stricto* in Japan, based on three pairs of primers designed to target the TRI6 gene region and one pair of primers designed to target the TRI3 gene [91]. Other *Fusarium* species including *F. acuminatum*, *F. avenaceum*, *F. culmorum*, *F. equiseti*, and *F. poae* tested in this study did not produce the expected PCR product for these primers.

#### 5.2.9. TRI7 and TRI13 in Combination

Genes TRI7 and TRI13 are key to the production of NIV trichothecene in sequential conversion steps from 3,15-acetyldeoxynivalenol to 3,15-diacetylnivalenol (3,15-diANIV) and then to 3,4,15-diANIV (Figure 3d,e) [16,41,66,83,92]. Lee et al. [66] reported on amplicon length difference between NIV and DON chemotype after gene-specific PCR amplification of the TRI7 gene. Both TRI7 and TRI13 genes encode enzymes in NIV-producing isolates, while in DON-producers, multiple mutations result in an inability to produce NIV [36,83]. Chandler et al. [83] reported on the development of PCR assays based on these two genes which accurately indicate a DON or NIV chemotype in *F. graminearum*, *F. culmorum* and *F. cerealis* and proposed that this data can be used to estimate the risk of trichothecene contamination. Starkey et al. [93] method proved to be effective on *F. culmorum* and *F. cerealis* based on a multiplex PCR assay to detect both TRI7 and TRI13 genes simultaneously. Mugrabi de Kuppler et al. [75] also described characterizing the TRI7 and TRI13 genotype as DON and NIV chemotypes using gene-specific primers for *F. graminearum* isolates from Germany; however, there was discordance with respect to the quantity of trichothecene produced and quantified by LC-MSMS analysis.

#### 5.2.10. TRI3 and TRI12 in Combination

Wei et al. [79] reported on PCR assays based on the TRI3 gene (Figure 3a,b) and on the TRI12 gene [58,89]. These primers target trichothecene B-producing *Fusarium* species and the size of the amplicon is indicative of the chemotype of the isolate (Table 3). A combination of both genes either singly or in multiplex PCR should be congruent for both TRI3 and TRI12 genes. TRI3 and TRI12 are located toward the 5′ and 3′ end of the TRI gene cluster and appear to be conserved among certain species of *Fusarium*. Combined PCR genotyping of TRI3–TRI12 polymorphisms has been touted as the most robust assay for correlating trichothecene chemotypes [46].

## 6. Molecular Detection of mRNA Does Not Generally Predict its Protein Level

Real-time PCR using TaqMan^®^ gene expression assays for detection and quantification of mRNA transcribed from TRI12 genes have been described [68,69]. A key assumption in studying mRNA expression is that the ratio of mRNA levels to protein levels remains constant across tissues for any given gene, i.e., the correlation is positive in systems existing in a steady state [94]. However, Fortelny et al. [95] debunked this proposal to predict protein abundance using “the median ratio of protein to mRNA levels as a proxy of the gene-specific translation rate constant” and reported that the proposed correlations significantly overestimate the accuracy of per-gene predictions. During highly dynamic conditions, such as during stress response, post-transcriptional, translational, post-translational processes, resource allocation and “buffering” mechanisms at the protein level can attenuate the production of mRNA. Living cells are hardly under steady state conditions. Liu et al. [96] concluded that transcript levels by themselves are inadequate in predicting production and/or levels of proteins in many scenarios, and thus cannot be used to explain genotype–phenotype relationships without additional verification.

## 7. Conclusion and Future Prospects

Establishing a direct nexus between TRI genotype, chemotype and toxicity of different classes of trichothecenes is not straightforward and validation of PCR assays should be carried out by chemical analysis of trichothecenes produced by each isolate. Regardless of the TRI genotype, TRI chemotype verification remains the purview of chemical analysis due to discordance at the intra- and interspecific levels and gene interactions, in addition to other factors. Nevertheless, these genotype, chemotype and toxicity characteristics should be studied in tandem to better understand TRI gene evolution, in addition to the risk of contamination and exposure by mycotoxin-producing *Fusarium* species. Identifying the evolutionary mechanisms that resulted in sorting, rearrangement and consolidation of TRI genes into the core gene cluster for some *Fusarium* species, and the possible exploitation of the complex mechanisms that regulate mycotoxin biosynthesis can assist in developing multi-tiered approaches for disease management. To date, the TRI gene cluster has been the primary target in developing molecular tools for detection and identification of genotypes and the potential corresponding chemotypes. This strategy has been successful for some TRI genes and for some *Fusarium* species but not others, and for some isolates of a particular species within a specific geographical region but not others [46]. Additionally, climate change has a significant impact on the emergence of toxigenic fungi and the induction of mycotoxin production depending on the fungal species [97,98,99]. For example, changing climatic conditions has caused fluctuations in the mycotoxin-producing populations of *Fusarium* species infecting wheat in Europe and resulted in increased infection and mycotoxin contamination by *F. graminearum* in Central and Northern Europe [100]. This can have potential geographical and temporal trichothecene genotype shifts in this region. Nielsen et al. [69] also reported on a possible chemotype–history relationship based on climate change. These effects are monitored as part of the MYCORED PROJECT (http://www.mycored.eu/) [101] which is a European-driven multinational framework detailing specific targets and objectives for 10 work packages whose objectives include developing novel integrated strategies to reduce contamination by mycotoxins of major concern in economically important food and feed chains. Work package No. 6 (WP6) “Advanced technologies for diagnostics, quantitative detection and novel approaches to control toxigenic fungi”, which focuses on studying “the toxigenic fungal biodiversity by using the developed molecular methods to monitor mycotoxin risks”, has been led by Dr. Antonio Moretti since 2009. Comparison of TRI gene and amino acid sequences from correctly identified *Fusarium* species will also reveal polymorphism “hot spots”, the types of polymorphisms and regions of nucleotide conservation in the core TRI gene cluster. Whole genome sequencing and functional genomics will explain expression and regulation of TRI genes by focusing on gene transcription, translation and protein–protein interactions under different environmental conditions and for different *Fusarium* species.

## Figures and Tables

**Table 1:** Gene functions of the TRI gene cluster based on the genome sequence NC_026475.1: *Fusarium graminearum* PH-1 isolate (assembly ASM24013v3) and based on GenBank annotations and annotations reported by Proctor et al. [26]. GenBank annotations are largely incomplete [44].

Gene ID	Locus Tag	Gene	Protein Annotation (GenBank)	Protein Annotation [26]
Gene ID: 23550835	FGSG_03532	TRI8	trichothecene 3-O-esterase	esterase
Gene ID: 23550836	FGSG_03533	TRI7	hypothetical protein	acetyl transferase
Gene ID: 23550837	FGSG_03534	TRI3	hypothetical protein	acetyl transferase
Gene ID: 23550838	FGSG_03535	TRI4	trichodiene oxygenase	cytochrome P450 monooxygenase
Gene ID: 23550839	FGSG_03536	TRI6	transcription factor	Zn_2_His_2_ transcription factor
Gene ID: 23550840	FGSG_03537	TRI5	trichodiene synthase	terpene synthase
Gene ID: 23550841	FGSG_03538	TRI10	transcription factor	transcriptional regulator
Gene ID: 23550842	FGSG_03539	TRI9	hypothetical protein	unknown
Gene ID: 23550843	FGSG_03540	TRI11	isotrichodermin C-15 hydroxylase	cytochrome P450 monooxygenase
Gene ID: 23550844	FGSG_03541	TRI12	trichothecene efflux pump	major facilitator superfamily transporter
Gene ID: 23550845	FGSG_03542	TRI13	hypothetical protein	cytochrome P450 monooxygenase
Gene ID: 23550846	FGSG_03543	TRI14	hypothetical protein	unknown
NA	NA	TRI16	NA	acyl transferase
NA	NA	TRI101	NA	acetyl transferase

**Table 2:** Comparison of nucleotide and amino acid sequence similarities for each TRI gene.

TRI Gene	Gene Function	Nucleotide (nt) Sequence % Similarity			Amino Acid (aa) Sequence % Similarity		
		nt Sequence Length ^1^	Among Species ^2^	Within Species	aa Sequence Length	Among Species	Within Species
TRI8	esterase	625; 802	89–100%/N = 21	99–100%/N = 4	267	76–100%	98–100%
TRI7	acetyl transferase	1045	94–100%/N = 19	98–100%/N = 18; 30	331	73–100%	98–100%
TRI3	acetyl transferase	664; 809	85–100%/N = 17	99–100%/N = 23; 4	170	85–100%	99–100%
TRI4	cytochrome P450 monooxygenase	1186; 1217	75–100/N = 13; 15	94–100%/N = 4	368	86–100%	98–100%
TRI6	Zn_2_His_2_ transcription factor	450	84–100%/N = 18	98–100%/N = 18; 30	600	87–100%	98–100%
TRI5	terpene synthase	284; 504	77–100%/N = 41	89–100%/N = 13; 16; 59	111	80–100%	98–100%
TRI10	transcriptional regulator		89–100%/N = 39		420	70–100%	98–100%
TRI9	unknown	***	***	***	***	***	***
TRI11	cytochrome P450 monooxygenase	334	89–100%/N = 39	94–100%/N = 33	147	90–100%	98–100%
TRI12	major facilitator superfamily transporter/efflux pump	1967	***	99–100%/N = 4	600	87–100%	97–100%
TRI13	cytochrome P450 monooxygenase	1010; 1060	92–100%/N = 13	99–100%/N = 4	315	76–100%	98–100%
TRI14	unknown	658; 660	75–100%/N = 41	97–100%/N = 4; 11; 8	202	89–100%	99–100%
TRI16	acyl transferase	1143	90–100%/N = 8	99–100%/N = 4	381	60–100%	99–100%
TRI101	acetyl transferase	925; 1244	96–100%/N = 17; 33	99–100%/N = 11; 8; 6	444	79–100%	99–100%
TRI1	cytochrome P450 monooxygenase	664; 1755	94–100%/N = 8	99–100%/N = 100	512	85–100%	98–100%

^1^ Refers to different nucleotide sequence lengths depending on the submitter. Different sequence lengths are separated by a semi-colon. ^2^ N refers to the number of isolates within a specific POPSET. In some cases, more than one POPSET was used for comparison and this is separated by a semi-colon. *** No sequence data were obtained for gene and/or amino acid sequences in GenBank.

**Table 3:** Commonly used primers designed against specific target genes of the TRI gene cluster for *Fusarium.*

Gene Target	Primer Name	Primer Sequence 5′ to 3′	Amplicon Length/bp	Target Species	Reference
TRI3	3_CONS ^1^	TGGCAAAGACTGGTTCAC		*F. graminearum, F. asiaticum*	[79]
TRI3	3_NIV_F	GTGCACAGAATATACGAGC	840	*F. graminearum*, *F. asiaticum*	[79]
TRI3	3_15ADON_F	ACTGACCCAAGCTGCCATC	610	*F. graminearum, F. asiaticum*	[79]
TRI3	3_3ADON_F	CGCATTGGCTAACACATG	243	*F. graminearum, F. asiaticum*	[79]
TRI12	12_CONS	CATGAGCATGGTGATGTC		*F. graminearum, F. asiaticum*	[79]
TRI12	12_NIV_F	TCTCCTCGTTGTATCTGG	840	*F. graminearum, F. asiaticum*	[79]
TRI12	12_15ADON_F	TACAGCGGTCGCAACTTC	670	*F. graminearum, F. asiaticum*	[79]
TRI12	12_3ADON_F	CTTTGGCAAGCCCGTGCA	410	*F. graminearum, F. asiaticum*	[79]
TRI3	Tri3F971	CATCATACTCGCTCTGCTG	708for 15-ADON producers only	*F. graminearum, F. culmorum, F. cerealis*	[80]
	Tri3F1325	GCATTGGCTAACACATGA	354for 3-ADON producers only	*F. graminearum, F. culmorum, F. cerealis*	[80]
	Tri3R1679	TT(A/G)TAGTTTGCA TCATT(A/G)TAG		*F. graminearum, F. culmorum, F. cerealis*	[80]
Gene F	3891	GCTGTCAYAGYCAGAAGYTACGATG	1200	*Fusarium incarnatum equiseti* species complex	[62]
	3894	AGAYATGBAGGACARGGCTTAGGGT		*Fusarium incarnatum equiseti* species complex	[62]
TRI1	1285	GCGTCTCAGCTTCATCAAGGCAKCKAMTGAWTCG	1200	*F. graminearum, F. sporotrichioides*	[30]
	1292	CTTGACTTSMTTGGCKGCAAAGAARCGACCA		*F. graminearum, F. sporotrichioides*	[30]
TRI3	1912	TGTGTMGGYGCWGAGGCVATYGTTGG		*F. graminearum, F. sporotrichioides*	[30]
	1914	ACRGCAGCRGTCTGRCACATGGCGTA		*F. graminearum, F. sporotrichioides*	[30]
TRI4	2576	CCAATCAGYCAYGCTRTTGGGATACTG	1800	*F. graminearum, F. sporotrichioides*	[30]
	2578	ACCCGGATTTCRCCAACATGCT		*F. graminearum, F. sporotrichioides*	[30]
TRI5	1558	GGCATGGTCGTGTACTCTTGGGTCAAGGT	1300	*F. graminearum, F. sporotrichioides*	[30]
	1559	GCCTGMYCAWAGAAYTTGCRGAACTT		*F. graminearum, F. sporotrichioides*	[30]
TRI8	3904	GACCAGNAYCACSGYCAACAGTTCAG	1200	*Fusarium incarnatum equiseti* species complex	[62]
	3906	GAACAGCCRCTCCRWAACTATTGTC		*Fusarium incarnatum equiseti* species complex	[62]
TRI11	3895	TWCCCCACAAGRAACAYCTYGARCT	1300	*Fusarium incarnatum equiseti* species complex	[62]
	3897	TCCCASACTGTYCTSGCMAGCATCAT		*Fusarium incarnatum equiseti* species complex	[62]
TRI16	1472b	CCTCTCTCCCCTTGAYCAATTRAACTCT	NA	*F. graminearum, F. sporotrichioides*	[30]
	1473b	CTTCCCGATCCCRAYGAGCCTCTTACAC		*F. graminearum, F. sporotrichioides*	[30]
	1474b	GCCTTATMTKGGTAATGTCGTGCTKACA		*F. graminearum, F. sporotrichioides*	[30]
	1475b	AAGAGGCTCRTYGGGATCGGGAAGGTTC		*F. graminearum, F. sporotrichioides*	[30]
	1476b	CARCCGACGATGTMAGCACGACATTACC		*F. graminearum, F. sporotrichioides*	[30]
	1477b	CAATATACGGATACCGCACAAAGACTGG		*F. graminearum, F. sporotrichioides*	[30]
TRI101 ^2^	109	CCATGGGTCGCRGGCCARGTSAA	NA	*F. graminearum, F. sporotrichioides*	[30]
	178	AACTCSCCRTCIGGYTTYTTNGGCAT		*F. graminearum, F. sporotrichioides*	[30]
TRI5	HATri/F	CAGATGGAGAACTGGATGGT	260	*F. culmorum, F. poae, F. sporotrichioides, F. graminearum, F. sambucinum*	[81]
	HATri/R	GCACAAGTGCCACGTGAC		*F. culmorum, F. poae, F. sporotrichioides, F. graminearum, F. sambucinum*	[81]
TRI11	N11	CTTGTCAGGCGGCACAGTAG	643 for NIV-producers	*F. asiaticum, F. mesoamericanum, F. cortaderiae, F. gerlachii, F. meridionale × F. asiaticum, F. meridionale, F. lunulosporum, F. cerealis, F. vorosii, F. aethiopicum, F. graminearum, F. boothii, F. asiaticum, F. brasilicum, F. austroamericanum, F. culmorum, F. pseudograminearum*	[74]
	15D11	AAGTATGGTCCAGTTGTCCGTATT	424 for 3-ADON producers	*F. asiaticum, F. mesoamericanum, F. cortaderiae, F. gerlachii, F. meridionale × F. asiaticu), F. meridionale, F. lunulosporum, F. cerealis, F. vorosii, F. aethiopicum, F. graminearum, F. boothii, F. asiaticum, F. brasilicum, F. austroamericanum, F. culmorum, F. pseudograminearum*	[74]
	3D11	GCAA GTCTGGCGAGGCC	342 for 15-ADON producers	*F. asiaticum, F. mesoamericanum, F. cortaderiae, F. gerlachii, F. meridionale × F. asiaticu), F. meridionale, F. lunulosporum, F. cerealis, F. vorosii, F. aethiopicum, F. graminearum, F. boothii, F. asiaticum, F. brasilicum, F. austroamericanum, F. culmorum, F. pseudograminearum*	[74]
	11R	TCAAAGGCCAGAGCA ACCC		*F. asiaticum, F. mesoamericanum, F. cortaderiae, F. gerlachii, F. meridionale × F. asiaticu), F. meridionale, F. lunulosporum, F. cerealis, F. vorosii, F. aethiopicum, F. graminearum, F. boothii, F. asiaticum, F. brasilicum, F. austroamericanum, F. culmorum, F. pseudograminearum*	[74]
TRI11	Tri11-CON	GACTGCTCATGGAGACGCTG	NA	*F. graminearum*	[82]
	Tri11-3AcDON	TCCTCATGCTCG GTGGACTCG	334	*F. graminearum*	[82]
	Tri11-15AcDON	TGGTCCAGT TGTCCGTATT	279	*F. graminearum*	[82]
	Tri11-NIV	GTAGGTTCCATTGC TTGTTC	497	*F. graminearum*	[82]
TRI7	GzTri7/f1	GGCTTTACGACTCCTCAACAATGG	∼160	*F. graminearum*	[66]
	GzTri7/r1	AGAGCCCTGCGAAAG(C/T)ACTGGTGC		*F. graminearum*	[66]
TRI7	Tri7F340	ATCGTGTACAAGGTTTACG	625	*F. graminearum, F. culmorum, F. cerealis*	[80]
	Tri7R965	TTCAAGTAACGTTCGACAAT		*F. graminearum, F. culmorum, F. cerealis*	[80]
TRI13	Tri13F	CATCATGAGACTTGTKCRGTTTGGG	1075 for NIV producers; 799 for DON-producers	*F. graminearum, F. culmorum, F. cerealis*	[83]
	Tri13DONR	GCTAGATCGATTGTTGCATTGAG	282 for DON producers	*F. graminearum, F. culmorum, F. cerealis*	[83]
	Tri13R	TTGAAAGCTCCAATGTCGTG		*F. graminearum, F. culmorum, F. cerealis*	[83]
	Tri13NIVF	CCAAATCCGAAAACCGCAG	312 for NIV producers	*F. graminearum, F. culmorum, F. cerealis*	[83]

^1^ Length of the amplicon after PCR in combination with the conserved primers 3_CONS or 12_CONS. ^2^ In nucleotide sequences, K = G or T, M = A or C, S = C or G, R = A or G, W = A or T, Y = C or T, and I = inosine.

## Supplementary Materials

The following are available online at http://www.mdpi.com/2072-6651/11/1/36/s1, Table S1: Hyperlinks to all PopSet data available in GenBank and that were used to determine intra- and interspecific trichothecene gene variation among different *Fusarium* species.
