# Supplementary material for: Selection of Fusarium Trichothecene Toxin Genes for Molecular Detection Depends on TRI Gene Cluster Organization and Gene Function
Source: Toxins (Basel). 2019 Jan 14;11(1):36. doi: 10.3390/toxins11010036 (PMC6357111; doi:10.3390/toxins11010036)
Supplement: Supplementary file 1 [file toxins-11-00036-s001.pdf]

Supplementary Materials: Selection of Fusarium Trichothecene Toxin Genes for Molecular Detection Depends on TRI Gene Cluster Organization and Gene Function

Ria T. Villafana, Amanda C. Ramdass and Sephra N. Rampersad

Table S1. Hyperlinks to each PopSet data available in GenBank and which were used to determine intra- and interspecific trichothecene gene variation among different *Fusarium* species.

TRI Gene	PopSet (Hyperlink)
TRI101	https://www.ncbi.nlm.nih.gov/popset/?term=fusarium++TRI101
TRI1	https://www.ncbi.nlm.nih.gov/popset/?term=fusarium+TRI1
TRI3	https://www.ncbi.nlm.nih.gov/popset/?term=fusarium+TRI3
TRI4	https://www.ncbi.nlm.nih.gov/popset/?term=fusarium+TRI4
TRI5	https://www.ncbi.nlm.nih.gov/popset/?term=fusarium+TRI5
TRI6	https://www.ncbi.nlm.nih.gov/popset/?term=fusarium+TRI6
TRI7	https://www.ncbi.nlm.nih.gov/popset/?term=fusarium+TRI7
TRI8	https://www.ncbi.nlm.nih.gov/popset/?term=fusarium+TRI8
TRI9	https://www.ncbi.nlm.nih.gov/popset/?term=fusarium+TRI9
TRI11	https://www.ncbi.nlm.nih.gov/popset/?term=fusarium+TRI11
TRI12	https://www.ncbi.nlm.nih.gov/popset/?term=fusarium+TRI12
TRI13	https://www.ncbi.nlm.nih.gov/popset/?term=fusarium+TRI13
TRI14	https://www.ncbi.nlm.nih.gov/popset/?term=fusarium+TRI14
TRI15	No PopSets deposited for this gene
TRI16	https://www.ncbi.nlm.nih.gov/popset/?term=fusarium+TRI16
